# Association of life course adiposity with risk of incident dementia: a prospective cohort study of 322,336 participants

**DOI:** 10.1002/alz.087219

**Published:** 2025-01-09

**Authors:** Deng Yue‐Ting

**Affiliations:** ^1^ Huashan hospital, Fudan University, Shanghai, Shanghai China

## Abstract

**Background:**

Cohort studies report inconsistent associations between body mass index (BMI) and all‐cause incident dementia. Furthermore, evidence on fat distribution and body composition measures are scarce and few studies estimated the association between early life adiposity and dementia risk.

**Method:**

322,336 individuals of European ancestry were included in the main analysis after exluding people with baseline dementia, who are younger than 50 years old and who are non‐white. All participants joined the UK Biobank study from 2006 to 2010 and were followed up until December 31, 2020. Overall adiposity was comprised of birthweight (BW), childhood body size and adulthood body mass index (BMI). Other adiposity measures included central adiposity, body composition and fat distribution. Incident all‐cause dementia, ascertained through hospital inpatient, death records, and primary care.

**Result:**

Among the 322,336 individuals (mean [SD] age, 62.24 [5.41] years; 53.9% women) in the study, during a median 8.74 years of follow‐up, 5083 all‐cause incident dementia events occurred. The risk of dementia was 22% higher with plumper childhood body size (*P* < 0.001). A strong U‐shaped association was observed between adult BMI and dementia. More fat and less fat‐free mass distribution on arms were associated with a higher risk of dementia. Interestingly, similar U‐shaped associations were found between BMI and four metabolites (i.e., 3‐hydroxybutrate, acetone, citrate and poly unsaturated fatty acids), four inflammatory cells (i.e., neutrophil, lymphocyte, monocyte and leukocyte) and abnormalities in brain structure that were also related to dementia.

**Conclusion:**

In this large cohort study, adiposity in both childhood and adulthood were significantly associated with risk of dementia and a strong U‐shaped association existed between BMI and dementia. The findings that adiposity is associated with metabolites, inflammatory cells and abnormalities in brain structure that were related to dementia risk might provide clues to underlying biological mechanisms. Interventions to prevent dementia should begin early in life and include not only BMI control but fat distribution and body composition.

